# A human rights-based approach to non-communicable diseases: mandating front-of-package warning labels

**DOI:** 10.1186/s12992-021-00734-z

**Published:** 2021-07-28

**Authors:** Andrés Constantin, Oscar A. Cabrera, Belén Ríos, Isabel Barbosa, Ariadna Tovar Ramírez, Margherita M. Cinà, Silvia Serrano Guzmán

**Affiliations:** Global Center for Legal Innovation on Food Environments at the O’Neill Institute for National and Global Health Law, Georgetown University, Washington, DC USA

**Keywords:** Unhealthy diets, Non-communicable diseases, Human rights, Front-of-package warning labeling

## Abstract

Across the globe, the consumption of energy-dense and nutrient-poor foods and beverages has escalated rates of diet-related non-communicable diseases (NCDs), driven by deceptive marketing tactics from the food and beverage industry. The international community has increasingly recognized the need to provide consumers with accurate health information on food and beverage products as part of their right to health. In July 2020, the U.N. Special Rapporteur on the right to health released a powerful Statement calling for the adoption of front-of-package warning labeling to tackle NCDs. Just a few weeks after the Statement’s release, the Pan American Health Organization published a report highlighting the relevance of front-of-package labeling as a policy tool for the prevention of NCDs in the Americas, demonstrating further support to this regulatory intervention.

In this piece, we explain why front-of-package warning labeling should be part of a comprehensive strategy to promote healthier lives, delving into the human-rights aspects of front-of-package labels. In particular, we explore the role the food and beverage industry play in increasing the consumption of unhealthy foods and beverages, and the relevance of scientific evidence free from conflicts of interest to adequately protect the right to health and health-related rights.

## Background

Overweight and obesity are increasing at alarming rates, leading to the rise of non-communicable diseases (NCDs), such as cardiovascular diseases, cancers, and diabetes worldwide [1, 2]. NCDs are the leading cause of global morbidity and mortality, accounting for over 40 million deaths per year and imposing significant societal and economic costs [1, 2]. NCDs also compound the burden of infectious diseases like COVID-19 on health systems, with hospitals around the world ill-equipped to meet surges in patients requiring attention from health systems [3]. As NCDs are largely attributable to modifiable risk factors like tobacco use, alcohol consumption, unhealthy diets, and physical inactivity, many of these diseases and associated deaths are preventable. In particular, the consumption of energy-dense and nutrient-poor food and beverage products containing excessive levels of critical nutrients, such as sugars, sodium, and fats, poses a high risk for obesity and diet-related NCDs [4–8]. Globally, 20% of all deaths are associated with poor diet [1].

Globalization has created near-universal access to unhealthy products with excessive critical nutrients, shifting traditional dietary patterns to increase the consumption of processed and ultra-processed foods and beverages [9]. This increase is driven by brand recognition, widespread availability, low cost, and advertising and marketing strategies of energy-dense and nutrient-poor foods across the global population [10]. In particular, the food and beverage industry often designs the packaging and labeling of unhealthy foods and beverages to attract persons to purchase and consume them [11]. In many instances, consumers are left with little truthful or easily comprehensible information on a product’s sugar, sodium, or fat content – putting them at higher risk of making uninformed choices that lead to overweight, obesity, and diet-related NCDs.

The international community has increasingly recognized the need to provide consumers with accurate information on food and beverage products as part of their right to health [12]. In 2013, the World Health Organization recommended a series of interventions, including the adoption of front-of-package labeling, to help prevent overweight, obesity, and diet-related NCDs by countering the food and beverages industry’s marketing tactics and assisting consumers in making healthier choices [13, 14]. Yet, despite WHO’s recommendation being almost a decade old, most governments have not acted upon it. A more recent action came from the former U.N. Special Rapporteur on the right of everyone to the enjoyment of the highest attainable standard of physical and mental health, Dr. Dainius Püras.

In July 2020, U.N. Special Rapporteur Dr. Püras released a powerful Statement calling for the adoption of front-of-package warning labeling to tackle NCDs [15]. The Statement characterized NCDs as “a major challenge of this century highly rooted on overweight, obesity and unhealthy diets,” and was endorsed by both the U.N. Special Rapporteur on the right to food and the U.N. Working Group on the issue of human rights and transnational corporations and other business enterprises. While previous U.N. groups and experts had urged States to regulate the food and beverage industry’s promotion of unhealthy food products, this Statement went further [8]. It singled out a concrete public health regulatory measure – front-of-package warning labeling – as an effective means for promoting healthier lives and protecting the public’s health. A few weeks after the Statement was released, the Pan American Health Organization published a report highlighting the relevance of front-of-package labeling as a policy tool for the prevention of NCDs in the Americas, manifesting further support by the international community to this regulatory intervention [16].

By indicating to consumers which products contain excessive levels of sugars, fats, or sodium, front-of-package labels can provide accurate, transparent, and comprehensible information to enable informed purchase and consumption choices [17–19]. Legal and regulatory interventions mandating front-of-package labeling have demonstrated effectiveness in providing information to consumers to make healthier choices and discouraging consumption of unhealthy foods and beverages [16].

Governments have employed a range of voluntary or mandatory front-of-package labeling schemes, such as endorsement systems (e.g., Sweden), health star ratings (e.g., Australia), traffic light symbols (e.g., Ecuador), or numerical guidelines for daily amounts (e.g., United Kingdom) [16]. However, comparative scientific studies have proven warning labeling to be the most effective system for consumers to clearly understand and identify unhealthy products, thus allowing them to make healthier decisions [20, 21]. Other labeling systems have been found less effective. Endorsement systems, which provide a limited amount of information about a positive attribute of a product, have been found inadequate at informing consumers about the healthfulness of a product [22, 23]. Informative systems, like Daily Guideline Amounts, provide a truncated version of the nutritional facts on the front of the package and have frequently been promoted by the food and beverage industry [24]. These systems have confused consumers, especially vulnerable populations that may lack the required reading and comprehension skills and have had little impact on consumer decisions [25]. Summary systems, which score products for overall healthfulness, have been found easy to comprehend but insufficient to inform consumers about the content of critical nutrients [25, 26]. Nutrient-specific color-coded systems (traffic-light systems) have similarly demonstrated minimal effect in informing consumer decisions, especially when compared to warning label systems [16, 27].

Front-of-package *warning* labeling schemes on products with excessive critical nutrients (e.g., Chile, Mexico, Israel, Peru, and Uruguay) are particularly effective to enable healthier choices “without major investment of time and cognitive effort … by clearly marking such excess with warning labels arranged on the front of the product” [16–19, 22, 26–29]. Scientific studies point to warning labeling as the most effective front-of-package labeling system to inform consumer decisions [18–23, 25–27, 29]. Warning labels inform consumers by placing a “HIGH IN” or “EXCESS” warning/stop sign for every critical nutrient that exceeds the accepted threshold [16]. Compared to other labeling schemes, studies have found warning labels to be the superior option for capturing consumers’ attention, being easy to understand among various populations, thus changing consumption patterns [18–23, 25–27, 29]. Real-world settings evaluations have confirmed the effectiveness of warning labels [28, 30, 31]. Just one month after Uruguay mandated warning labels, 77% of consumers reported they had noticed the warning labels when making food purchases, and 58% reported modifying their purchasing decisions after seeing the warning [31]. In Chile, front-of-package warning labeling led to a decrease of nearly 24% in the purchase of sugar-sweetened beverages in the years following implementation [30, 32]. According to Chile’s Ministry of Health evaluations, over 90% of persons surveyed reported that the labels were comprehensible, and 68% reported using the labels to change consumption habits [28]. Compared to other front-of-package labeling systems, the superiority of warning labels is likely attributable to its design simplicity: a mono-color (usually black) stop sign with simple text informs and alerts the consumer without causing sensory or information overload [16].

The food and beverage industry strongly opposes front-of-package warning labeling systems, and has put forth baseless arguments as to why they should not be implemented, including the negative impacts on trade, high costs of implementation, and consumer responsibility to make educated consumption decisions [24]. While there is scant evidence supporting these arguments, the real downside of implementing front-of-package labeling systems derives from the industry opposition itself [24, 33]. Governments must have an enormous amount of time, resources, and political will to overcome well-funded and coordinated industry opposition tactics [24, 33]. Furthermore, it takes a robust legal scheme to enforce front-of-package labeling requirements once adopted. Where the food and beverage industry succeeds in dismantling any aspect of that scheme, the effectiveness of the labeling in yielding healthier consumer decisions could be diminished [24, 33]. Still, several countries have demonstrated that overcoming industry opposition is both possible and worthwhile for supporting the right to health through informed consumer decisions [30, 31].

In what follows, we explain why front-of-package warning labeling should be part of a comprehensive strategy to promote healthier lives, delving into the human rights aspects of front-of-package labels. In particular, we explore the role the food and beverage industry plays in the increasing consumption of unhealthy foods and beverages, and the relevance of scientific evidence behind front-of-package warning labeling to adequately protect the right to health and health-related rights.

## Main text

### Front-of-package warning labeling to support the right to health

Mandating the implementation of front-of-package warning labeling supports the right to health. By requiring accurate and reliable information regarding diet-related NCD risk factors, it allows consumers to make informed decisions, thus countering the food and beverage industry’s deceptive marketing tactics [17–19, 22, 26–29]. The logic is as follows: when consumers see warning labels with comprehensible information, the warnings affect their purchasing decisions, discouraging the consumption of foods and beverages with excessive critical nutrients associated with NCDs. Ultimately, changes in foods purchased will lead to overall dietary changes across the population. Numerous studies have indicated that dietary changes (i.e., decreased consumption of critical nutrients), in turn, contribute to reductions in overweight, obesity, and diet-related NCDs [31]. By empowering consumers with the information to make healthier decisions for themselves and their families, warning labels increase the standard of attainable health.

In the context of diet-related NCDs, the right to health imposes three primary obligations on States: to protect, respect, and fulfill [15]. Under international human rights law, States’ obligation to protect the right to health includes preventing interference with said right by non-State actors, such as corporations subject to their jurisdiction [15]. The food and beverage industry interfere with the right to health when they convey inaccurate, deceptive, or misleading information on unhealthy products to encourage their consumption [11]. When designing the packaging and labeling of their products, the food and beverage industry exploits consumers’ cognitive biases by explicitly targeting subconscious processes, attracting consumers to unhealthy products through colors, pictures, shapes, and designs [11]. Other marketing strategies are more directly misleading or deceptive on nutritional content. For example, they often place “claims” on packaging (e.g., “high in protein”) to mislead consumers into believing the product has an overall nutritious content – distracting the consumer from unhealthy levels of sodium, sugars, or fats [34].

In turn, the State’s failure to regulate the food and beverage industry’s activities to prevent them from interfering with the right to health entails a violation of the obligation to protect these rights. The obligation to protect requires direct regulation and intervention to restrict marketing and advertising (including on packaging) of unhealthy foods and beverages with excessive amounts of critical nutrients [15].

In part, States can meet their obligation to protect the right to health by mandating front-of-package warning labeling on unhealthy food and beverage products, thus countering the food and beverage industry’s interference with these rights [15]. Front-of-package warning labeling has proven efficacy in allowing consumers to clearly and effectively identify products with a nutritional profile detrimental to health and modify their purchasing decisions accordingly [17–19, 22, 26–29]. Ultimately, this labeling system “reduces the perception of healthfulness of certain food products that contain excessive levels of critical nutrients, such as sugars, sodium, total fats, trans-fats, and saturated fats among consumers.” [15] Thereby, it “promotes healthy decisions, discourages the consumption of [these] food products…, and counteracts the effects of living in an obesogenic environment” [15, 17–19, 22, 26–29].

Similarly, States’ obligation to fulfill the right to health requires them to actively adopt measures to provide the public with appropriate information and encourage informed decision-making on nutritional matters [15]. Mandating front-of-package warning labels effectuates this obligation by alerting consumers to products with excessive levels of sodium, sugars, and fats to promote healthy decisions [17]. In addition, the “warning” component of this labeling system promotes equality among consumers by guaranteeing access to comprehensible information on health across the population [16]. While States must also “improve the availability and accessibility of healthy foods,” front-of-package warning labels remain critical to ensuring consumers can differentiate between nutritious products and those likely to contribute to diet-related NCDs [15].

Furthermore, States’ obligation to respect the right to health in the context of unhealthy diets requires them “not to engage in any conduct that is likely to result in preventable morbidity or mortality, including incentivizing the consumption of unhealthy foods and beverages.” [15] This obligation also requires States to refrain from partially or fully suspending public health measures likely to protect people’s health. Where States have adopted measures to mandate front-of-package warning labeling, any decision to suspend or rescind the measure in question (especially when sparked by the food and beverage industry’s opposition) may constitute a violation of the State’s obligation to respect the right to health.

With international human rights law imposing specific obligations on States, front-of-package warning labeling stands out as an effective intervention for States to meet said obligations under the right-to-health framework. However, the adoption of front-of-package warning labeling is just one “key component of a comprehensive strategy to promote healthier lives.” By helping define the universe of unhealthy foods and beverages, it is “an effective stepping stone for States to pursue a set of additional measures that promote and protect the right to health, such as taxation, regulating school environments, and imposing marketing restrictions.” [15] Consequently, States should not limit their actions to address diet-related NCDs risk factors to adopting a front-of-package warning labeling system. Instead, they should include it as part of a robust plan to reduce the consumption of unhealthy foods and beverages through the use of a broader set of laws and regulations [35].

### The food and beverages industry’s influence on government decision-making

Governments have sought to adopt and implement front-of-package labeling and other regulatory strategies to support the right to health. However, they have often faced pervasive, organized, and effective opposition from the food and beverage industry. As such, former U.N. Special Rapporteur Dr. Püras called on States to “decisively counter undue influence of corporations on government decision-making by strengthening legal frameworks and safeguard the policies that protect the right to health, such as the front-of-package warning labeling, from commercial and other vested interests.” [15] The food and beverage industry has posed a direct threat to the right to health by employing a range of tactics to influence, prevent, or postpone government decision-making processes aimed at protecting public health [36].

This industry has engaged in multiple tactics to interfere and influence the adoption of public health measures, including spreading misinformation on the health and economic impacts of public health measures, initiating or threatening litigation, promoting weaker alternatives, and contesting the legality of such measures under international trade law [36, 37]. For example, the food industry met Chile’s front-of-package labeling law with intense controversy over the compatibility of the law with World Trade Organization agreements [33]. Even though the Chilean government overcame this barrier, it nevertheless caused significant delays in its efforts to adopt a front-of-package warning labeling system [33]. Similarly, during the process leading up to Colombia’s announcement of the adoption of a front-of-package warning labeling system in 2020, the food and beverage industry strongly promoted their preferred alternative (a Daily Guideline Amount, a system found to be less effective at informing consumer decisions), alleged that the warning labels would violate international norms under the Codex Alimentarius (i.e., international standards relating to food), and falsely claimed that there was no evidence that the warning labels would benefit public health [24].

In addition to these overt tactics, the food and beverage industry simultaneously employs opposition strategies to front-of-package labeling that are less discernable to the general public. For instance, the food and beverage industry commonly seeks to influence the adoption of public health regulatory measures by co-opting regulators, that is, helping elect and supporting those in positions of power that will further the industry’s interests [38, 39]. They use their financial positions to donate directly and via political parties, thus securing politicians’ advocacy against potentially restrictive regulations. Moreover, they finance shadow groups designed to promote “grassroots” opposition to unfavorable regulations by employing duplicitous tactics. This strategy may be particularly effective, as the public is likely to perceive such groups as under-funded advocacy coalitions concerned with stopping potentially harmful or overreaching regulations. Furthermore, they use corporate social responsibility (CSR) platforms as a means of influencing politicians and governments in countries where they wish to develop a market further [40, 41]. They employ tactics under the guise of CSR to legitimize their activities and build goodwill with government agents.

Colombia’s work toward adopting front-of-package warning labels illustrates many of these discrete tactics [24]. The food industry formed alliances with media outlets, community organizations, and health organizations through a public-private partnership allegedly focused on addressing childhood nutrition. They also attempted to influence policymakers through lobbying and donations. Furthermore, the food industry tried to gain a foothold through former members of large trade associations who came to hold decision-making positions within the Colombian government [24].

In light of the wide range of tactics the food and beverage industry deploys to interfere with governments’ public health decision-making processes, it is particularly relevant for States to consider that international human rights law promotes rational and rigorous policymaking based on evidence free from conflicts of interest. The U.N. Special Rapporteur’s Statement explicitly acknowledged that the food and beverage industry sponsors research to downplay links to negative health impacts from their products, thus “covering up the harmful effects of their food products.” [15] Industry-funded research should be scrutinized as an intentional effort to cover up their products’ harm, and governments must be extremely cautious about allowing this research to inform their decision-making processes [36]. Evidence free from conflicts of interest in the development of public health measures is critical for the realization of human rights. In fact, the right to enjoy the benefits of scientific progress and its applications requires States to align public policies with the best available scientific evidence [42]. It is thus imperative that States take measures to avoid the risks associated with conflicts of interest in the adoption and implementation of public health policies such as front-of-package warning labeling [42]. Only then will these policies be designed to achieve their public health objective.

Scientific evidence free from conflicts of interest is also critical for determining the nutrient profile that will inform the threshold over which a particular food or beverage product will require the front-of-package warning label. Nutrient profiling, or “the science of classifying or ranking foods according to their nutritional composition… to [prevent] disease and [promote] health,” is a complex technical matter that exceeds the scope of this article [43]. However, it is crucial to understand that the chosen nutrient profile will determine which products contain warning labels. Thus, its determination must be based on sound scientific evidence to achieve the public health goal [44].

Scientific evidence is crucial in policymaking, and front-of-package warning labeling is no exception. A given regulatory measure is only effective inasmuch as it contributes to achieve the desired outcomes, which only scientific evidence free from conflicts of interest can determine.

### The evolving science behind food and beverage labeling and health

The public health goal of front-of-package warning labeling is to discourage the consumption of unhealthy foods and beverages that contribute to the development of diet-related NCDs by informing consumers’ decisions based on the products’ nutritional content [16]. There is established scientific evidence on how governments can design and implement warning labels to be most effective at achieving this goal [17–23, 25–31]. Importantly, front-of-package warning labels should be mandatory. Studies have found that voluntary systems create inconsistencies among labeling practices that confuse consumers [17].

As States craft public policies to protect public health, it is important to bear in mind that the food and beverage industry will not remain passive in the face of stricter regulatory measures. Instead, they are known to adjust their promotional strategies to increase the appeal or consumption of their unhealthy products. Therefore, States must continually monitor and evaluate their front-of-package labeling measures to “assess their impact as well as to identify where improvements are needed.” [15]

## Conclusion

The rise of diet-related NCDs is preventable. Adopting and implementing cost-effective regulatory interventions can help curb NCD rates. Front-of-package warning labeling represents a significant measure for States to prevent NCDs, therefore protecting the right to health and health-related rights.

The former U.N. Special Rapporteur on the right to health’s Statement constitutes a call for action. It sends a clear message that States must adopt regulatory measures aimed at tackling NCDs, singling out front-of-package warning labeling on unhealthy foods and beverages as one such measure. To effectively combat the growing burden of NCDs, governments must equip all people with the resources and information to make healthy decisions and avoid preventable risk factors that lead to premature morbidity and mortality.

COVID-19 has underscored the need for NCD prevention to be at the center of global health action. As the pandemic has exposed, our health care systems are not equipped to deal with an increased prevalence of diseases. Adopting public health regulatory measures that prevent diseases, such as front-of-package warning labeling, is crucial to protect the right to health and health-related rights. In a post-pandemic world, governments are especially pressed to consider adopting measures to improve diets and reduce the growing number of NCDs. Doing so will uphold human rights, protect public health, and free up health care resources to better respond to future infectious disease outbreaks.

## Data Availability

Not applicable.
